# Hibiscus acid and hydroxycitric acid dimethyl esters from Hibiscus flowers induce production of dithiolopyrrolone antibiotics by *Streptomyces* Strain MBN2-2

**DOI:** 10.1007/s13659-024-00460-0

**Published:** 2024-07-03

**Authors:** Felaine Anne Sumang, Alan Ward, Jeff Errington, Yousef Dashti

**Affiliations:** 1 Faculty of Medicine and Health, University of Sydney, Sydney, NSW 2015 Australia; 2 School of Biology, Newcastle University, Newcastle Upon Tyne, UK; 3 Sydney Infectious Diseases Institute, University of Sydney, Sydney, NSW 2015 Australia

**Keywords:** Plant–microbe interactions, Cryptic biosynthetic gene cluster, *Streptomyces*, Microbial natural products

## Abstract

Plants and microbes are closely associated with each other in their ecological niches. Much has been studied about plant–microbe interactions, but little is known about the effect of phytochemicals on microbes at the molecular level. To access the products of cryptic biosynthetic gene clusters in bacteria, we incorporated an organic extract of hibiscus flowers into the culture media of different Actinobacteria isolated from plant rhizospheres. This approach led to the production of broad-spectrum dithiolopyrrolone (DTP) antibiotics, thiolutin (**1**) and aureothricin (**2**), by *Streptomyces* sp. MBN2-2. The compounds from the hibiscus extract responsible for triggering the production of these two DTPs were found to be hibiscus acid dimethyl ester (**3**) and hydroxycitric acid 1,3-dimethyl ester (**4**). It was subsequently found that the addition of either Fe^2+^ or Fe^3+^ to culture media induced the production of **1** and **2**. The Chrome Azurol S (CAS) assay revealed that **3** and **4** can chelate iron, and therefore, the mechanism leading to the production of thiolutin and aureothricin appears to be related to changes in iron concentration levels. This work supports the idea that phytochemicals can be used to activate the production of cryptic microbial biosynthetic gene clusters and further understand plant–microbe interactions.

**Graphical Abstract:**

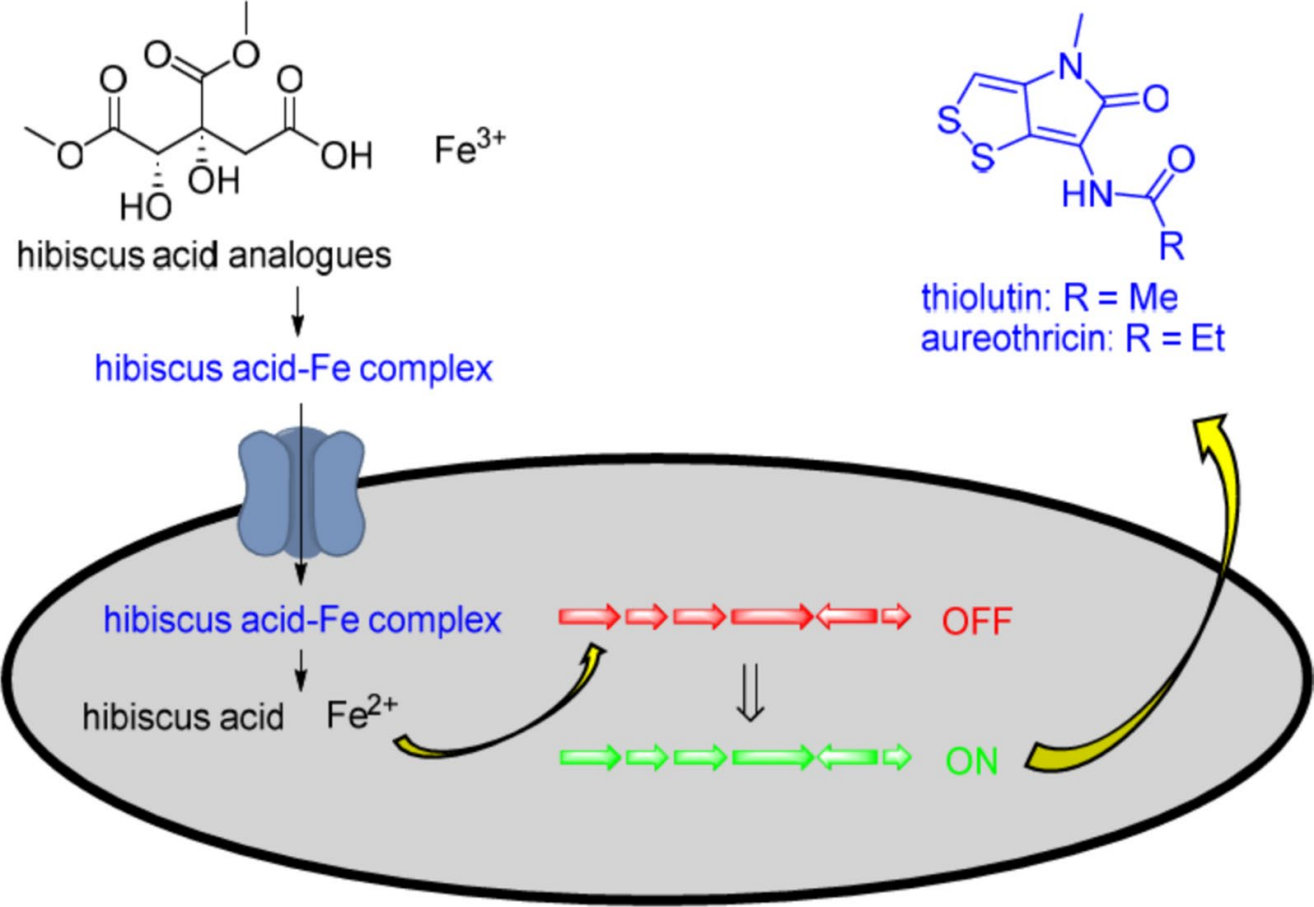

**Supplementary Information:**

The online version contains supplementary material available at 10.1007/s13659-024-00460-0.

## Introduction

Plant–microbe interactions in the rhizosphere has been a focal point of many ecological studies [1]. These interactions can be mutually beneficial or potentially harmful. In the realm of resource exchange, plants contribute significantly by supplying microbes with essential components such as carbon in form of sugar [2]. In return, microbes play a crucial role in converting organically-bound nitrogen, phosphorus, and sulphur in the soil into bioavailable forms, facilitating optimal nutrient absorption by plants through mineralization [2]. Other examples include heterotrophic bacteria that have the ability to convert atmospheric nitrogen into ammonia through enzymatic processes catalysed by nitrogenase [3]; bacteria that produce hydrolytic enzymes crucial for breaking down insoluble organic polymers into simpler forms; and siderophore producing microbes that aid in scavenging iron and other essential minerals. Ultimately, these nutrients become available for plants to utilize [4, 5]. To make the most of this relationship, plants have developed strategies such as releasing molecules like flavonoids to attract beneficial microbes, while concurrently deterring phytopathogens, thus shaping their root microbiome [6].

Organisms from the phylum Actinomycetota are common inhabitants of the soil and rhizosphere, and frequently can synthesise a wide range of specialised metabolites that are thought to be involved in adapting the organisms to their highly competitive environment, including symbiotic interactions with plants in the rhizosphere [5, 7–9]. For example, a specific category of bacteria known as plant growth-promoting rhizobacteria exhibits the capability to synthesize compounds like indole-3-acetic acid and gibberellic acid, which play pivotal roles in enhancing plant growth [10].

It is now well established that the genomes of the majority of Actinomycetota harbor numerous biosynthetic gene clusters (BGCs) that are either not expressed or expressed poorly under conventional laboratory culture conditions [11, 12]. One approach used to induce the production of compounds encoded by these cryptic BGCs is to mimic natural environments by co-cultivating two different Actinomycetota strains or an Actinomycetota strain with a fungal counterpart. This method has successfully led to the production of compounds that are not generated in independent cultures [13]. Taking inspiration from the interactions between plants and microbes, we aimed to mimic this phenomenon and investigate plant extracts as elicitors to induce the production of specialized metabolites from Actinomycetota strains isolated from plant rhizospheres. Here, we present our findings that hibiscus acid dimethyl ester (**3**) and hydroxycitric acid 1,3-dimethyl ester (**4**) from hibiscus induce production of the broad-spectrum antibiotics thiolutin (**1**) and aureothricin (**2**).

## Results and discussion

**Fig. 1:**
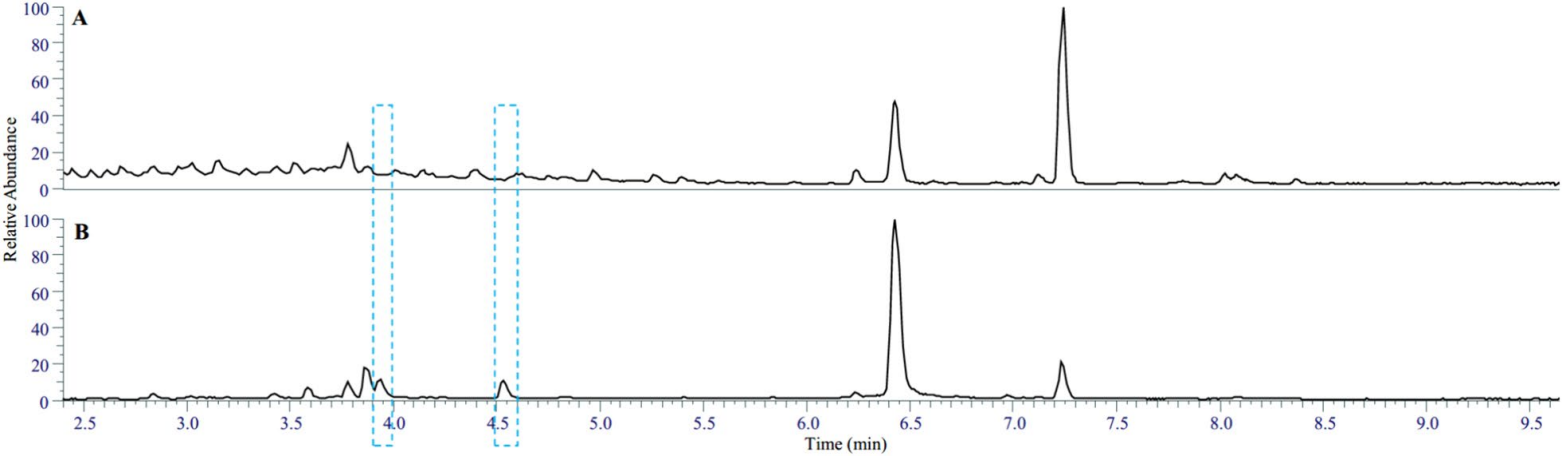
LC-HR-MS profile of culture supernatant of *Streptomyces* strain MBN2-2 grown **A** without and **B** with extract of hibiscus flower added to the culture media

**Fig. 2:**
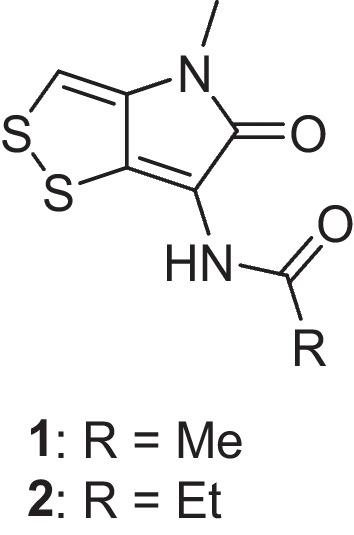
Structure of thiolutin (**1**) and aureothricin (**2**) produced by *Streptomyces* strain MBN2-2 in presence of the extract of hibiscus flower in culture media

For this induction study, 24 actinobacteria isolated from plant rhizosphere environments were selected. Plant extracts prepared from various plant parts were used as elicitors. These included hibiscus flowers, sage leaves, cinnamon, coriander seed, turmeric, and musk root. The extracts were tested at two final concentrations of 4.5 and 18 mg/mL, by adding them to overnight cultures of actinobacteria in YEME liquid medium. After seven days of incubation, 1 mL of the cultures was centrifuged, and the supernatants were subjected to analysis by LC–MS. Concurrently, their antimicrobial activity against various microorganisms including *Escherichia coli*, *Bacillus subtilis*, *Staphylococcus aureus*, *Cryptococcus neoformans*, and *Saccharomyces cerevisiae* was assessed. The culture supernatant of one *Streptomyces* isolate, designated as MBN2-2, exhibited significant antimicrobial activity against all tested microorganisms when cultivated in the presence of hibiscus extract (Figs. S1 and S2). This antimicrobial activity was absent when the strain was cultured without hibiscus extract. Comparison of the LC-HR-MS chemical profiles of these two cultures revealed two distinctive peaks in the profile of MBN2-2 cultured with hibiscus extract, which were absent in the non-treated culture (Fig. 1). These peaks corresponded to [M + H]^+^ masses at *m/z* 229.0100 and 243.0257 (Fig. S3), indicating molecular formulae of C_8_H_8_N_2_O_2_S_2_ and C_9_H_10_N_2_O_2_S_2_, respectively. Database searches identified thiolutin and aureothricin, respectively, as likely candidates for the active molecules (Fig. 2).

To substantiate the above observations the *Streptomyces* strain MBN2-2 was cultured at large scale (250 mL in YEME medium) with and without hibiscus extract. After seven days of incubation, ethyl acetate extracts were prepared from the cultures and subjected to semipreparative HPLC fractionation. A bioassay-guided approach was employed to identify the active fractions. Upon pairwise comparison of the HPLC fractions derived from the cultures with and without hibiscus extract, it was found that fractions 23 and 26 from the extract obtained in the presence of hibiscus exhibited an induced antimicrobial activity. LC-HR-MS analysis confirmed that these fractions contained compounds with masses corresponding to thiolutin and aureothricin, respectively. Subsequent verification of their structures was achieved through NMR spectroscopy (Figs. S4–S6). We then sequenced the genome of MBN2-2, and bioinformatics analysis revealed the presence of a gene cluster capable of supporting the biosynthesis of thiolutin and aureothricin (GenBank accession number: PP747228). Both thiolutin and aureothricin belong to a class of natural products distinguished by a unique bicyclic structure containing a dithiolopyrrolone ring system consists of a five-membered pyrrolone ring fused with a five-membered ring containing two sulfur atoms [14]. Dithiolopyrrolones have been isolated from various bacteria, including *Streptomyces albus* [15], *Saccharothrix algeriensis* [16], *Xenorhabdus bovienii* [17]*,* and *Alteromonas rava* [18]*.* This class of compounds are known for their wide range of activities against several human pathogens, including *S. aureus*, *E. coli*, *Klebsiella pneumoniae*, *Listeria monocytogenes*, *Candida albicans*, *Aspergillus carbonarius*, and *Fusarium culmorum* [14, 16, 19]. They also have broad-spectrum activity against phytopathogenic bacteria and fungi; for example, compounds **1** and **2** have shown strong activity against *Erwinia amylovora*, a bacterium that is responsible for apple fire blight [20]. Furthermore, in in vivo experiments they have been effective in suppressing tomato bacterial wilt and apple fire blight [20]. The antibiotic mode of action of thiolutin is attributed to interruption of diverse cellular pathways [21]. It is suggested that the disulfide bond of the thiolutin is reduced in the cell and subsequently chelates Zn^2+^, leading to inhibition of multiple metalloproteins. The reduced form also interacts with Mn^2+^ to inhibit transcription initiation by RNA polymerase II (Pol II) [22].

**Fig. 3:**
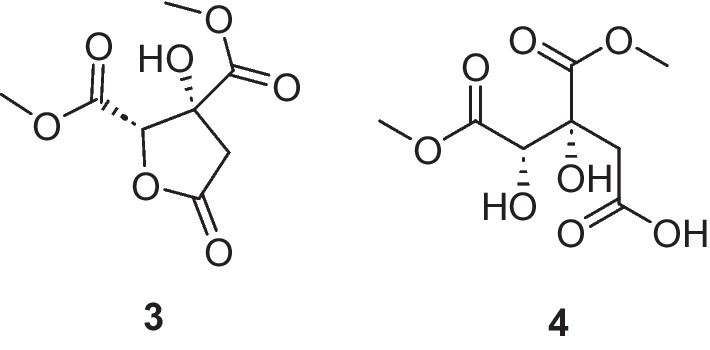
Structure of elicitors hibiscus acid dimethyl ester (**3**) and hydroxycitric acid 1,3-dimethyl ester (**4**) identified in the extract of hibiscus flower

Our next objective was to identify the compound/s from the hibiscus extract that were responsible for triggering thiolutin and aureothricin production. A methanolic extract of hibiscus flower was fractionated via semipreparative HPLC. The dried fractions were then resuspended in DMSO and added to overnight cultures of MBN2-2. Analysis of the cultures after seven days revealed that fractions 8 and 11 induced thiolutin and aureothricin production. LC-HR-MS analysis of these fractions revealed [M + H]^+^ peaks at *m/z* 219.0499 (C_8_H_11_O_7_) and 237.0604 (C_8_H_13_O_8_), potentially corresponding to hibiscus acid dimethyl ester (**3**) and hydroxycitric acid 1,3-dimethyl ester (**4**), respectively (Fig. 3). The identities of these compounds were confirmed by ^1^H NMR spectroscopy (Figs. S7 and S8). While it has been reported that plant exudates can contain citric acid and that this can enhance the colonization of beneficial microbes in the rhizosphere [23], we are not aware of reports that citric acid or related compounds such as hydroxycitric acid or its methyl esters, can induce the production of specialized metabolites in actinobacteria. To explore this possibility, a similar induction study was conducted using citric acid and hydroxycitric acid. LC-HR-MS profiling and antibacterial activity assays indeed confirmed the production of thiolutin and aureothricin induced by both compounds.

How thiolutin and aureothricin are induced by the addition of hibiscus acid dimethyl ester, hydroxycitric acid dimethyl ester, citric acid, and hydroxycitric acid remains unclear. Citric acid is recognized as an iron chelator, and various citrate-containing metabolites, such as staphyloferrin B, achromobactin, and vibrioferrin, have been recognised as siderophores produced by bacteria for iron acquisition [24, 25]. Hence, we speculated that iron availability might have a role in triggering the production of thiolutin and aureothricin. Previous studies have demonstrated that iron starvation can activate certain BGCs [26–28]. Additionally, a single biosynthetic pathway that can produce bagremycins under iron depletion conditions, also produces ferroverdins when iron is abundant [29]. Here, it seemed possible that citrate, hydroxycitrate, and related analogues might chelate free iron ions present in the medium, leading to iron starvation and consequent induction of thiolutin and aureothricin production. Alternatively, the complexes formed by these compounds with iron could be taken up by bacterial transporter systems supporting the activity of enzymes requiring iron for their function.

A Chrome Azurol S (CAS) assay was used to test whether hibiscus acid dimethyl ester, hydroxycitric acid 1,3-dimethyl ester, and hydroxycitric acid can chelate iron. As shown in Fig. S9, all compounds tested can chelate iron, albeit less avidly than citric acid. We then tested whether iron alone could induce thiolutin and aureothricin production. Compounds **1** and **2** were both made when either Fe^2+^ (iron (II) sulphate), or Fe^3+^ (iron (III) chloride) were added to a culture of *Streptomyces* strain MBN2-2, at a concentration of 0.75 mg/mL (Fig. S10), confirming the direct involvement of iron in triggering their biosynthesis. Therefore, the most plausible mechanism for the induced production of dithiopyrrolones **1** and **2** by *Streptomyces* strain MBN2-2 involves the iron chelation ability of compounds **3** and **4**. These compounds form complexes with iron, which are then transferred to the bacterial cytoplasm by siderophore-iron complex transporter systems. In the cytoplasm, the iron is released and becomes available for enzymes that require iron for their function.

## Conclusion

Understanding the interactions between microbe and plant at molecular level is of great importance. Several microbial specialized metabolites have been identified to be beneficial for plants through their insecticide (e.g. spinosad and abamectin) and fungicide activity (e.g. Burkholdines) [30, 31], or by promoting plant growth (e.g. indole-3-acetic acid and gibberellic acid) [10]. Considering that these interactions should be reciprocal, we tried to assess the effects of plant extracts on the production of specialized metabolites by *Actinomycetota* strains isolated from the plant rhizosphere. This led to identification of hibiscus acid dimethyl ester and hydroxycitric acid 1,3-dimethyl ester from hibiscus flowers as elicitors that induce production of the broad-spectrum antibiotics thiolutin and aureothricin by *Streptomyces* strain MBN2-2. To our knowledge, there are no previous reports on using plant extracts to induce production of cryptic BGCs in *Actinomycetota*.

## Materials and methods

### General experimental procedure

LC-HR-MS analysis was performed on Thermo Vanquish UHPLC connected to a Proshell 120 EC-C18 column (2.1 × 100 mm, 1.9 μm) linked to an Orbitrap IQ-X Tribrid mass spectrometer. NMR spectra were recorded on a Bruker 600 MHz spectrometer equipped with a TCI cryoprobe at 25 °C. The ^1^H and ^13^C NMR chemical shifts were referenced to the DMSO-*d*_*6*_ solvent peaks at *δ*_H_ 2.50 and *δ*_C_ 39.52. All HPLC and LC–MS experiments were performed with a MeCN-H_2_O gradient solvent system.

### Screening of plant extracts against Actinobacteria and antimicrobial activity assay

Hibiscus and other plant extracts were prepared by soaking 10 g of crushed dried plant parts in approximately 100 ml methanol with stirring for 1 h, then filtered and dried using a rotary evaporator. Stock solution of 900 and 225 mg/mL in DMSO were made for addition to Actinobacterial cultures. A total of 24 isolates were grown in 1.5 ml YEME liquid (3 g/L yeast extract, 3 g/L malt extract, 10 g/L glucose, 5 g/L peptone) in 12 well plates in three replicates. After 24 h, 30 µL of stocks of 900 and 225 mg/mL solutions of plant extracts were added to the culture to give final concentrations of 18 and 4.5 mg/mL, respectively. The cultures were incubated at 30 °C with shaking at 150 rpm for 7 days. For LC–MS profiling and antimicrobial activity assays,  1 mL of each culture was centrifuged for 5 min at 14,000 rpm. Supernatants were directly analysed by LC–MS (2 µL per injection) and disc diffusion assay (20 µL per each disc) against *B. subtilis, E. coli, S. aureus, C. albicans* and *C. neoformans*. Nutrient agar (NA) was used for all strains except *C. neoformans*, for which YPD (10 g/L yeast extract, 20 g/L peptone, 20 g/L glucose) was used.

### HPLC fractionation/purification of thiolutin and aureothricin

To purify induced metabolites, strain MBN2-2 was grown in 250 ml liquid YEME with and without 4.5 mg/ml hibiscus flowers extract for 7 days at 30 °C with shaking at 150 rpm. Cultures were then centrifuged, and the supernatants were filtered (Whatman™ no. 1). The filtrate was passed through a C18 based cartridge (Thermo Hypersep C18 10 G) using flash chromatography. Initially it was washed with two volumes of deionized water, then the extract was eluted with two volumes of methanol. The methanol was evaporated by use of a Genevac, then the dried extract was resuspended in 1 ml methanol, loaded onto dental cotton and air-dried in a fume hood. The dental cotton containing the extract was loaded into a cartridge (10 × 30 mm) connected to a semi-preparative reverse-phase C18 Betasil column (21.2 mm × 150 mm). An Agilent 1260 Infinity II preparative HPLC was used for fractionation of the extract using the following method: initially, constant 5% acetonitrile for 5 min, followed by a linear gradient from 5 to 95% acetonitrile for 55 min, then isocratic at 100% acetonitrile for the next 5 min at a flow rate of 9 min/ml. The fraction collector was set to collect fractions at 60-s intervals throughout a 60-min runtime. Fractions were assayed against *B. subtilis* and active fractions analysed by LC–MS and NMR.

Thiolutin (**1**): ^1^H NMR [DMSO-*d*_*6*_]: 9.98 (1H, s), 7.35 (1H, s), 3.25 (3H, s), 2.02 (3H, s). ^13^C NMR [DMSO-*d*_*6*_]: 168.9, 166.2, 136.0, 132.4, 114.8, 111.0, 27.5, 22.4. HRESIMS [M + H]^+^ *m/z* 229.0100 (calcd for C_8_H_9_N_2_O_2_S_2_, 229.0100).

Aureothricin (**2**): ^1^H NMR [DMSO-*d*_*6*_]: 9.90 (1H, s), 7.33 (1H, s), 3.25 (3H, s), 2.35 (2H, q, *J* = 7.5 Hz), 1.01 (3H, t, *J* = 7.6 Hz). HRESIMS [M + H]^+^ *m/z* 243.0257 (calcd for C_9_H_11_N_2_O_2_S_2_, 243.0256).

### Identification of elicitor compound from *Hibiscus* extract

Ten grams of crushed Hibiscus flowers was extracted with methanol for 1 h. Methanol was evaporated by rotary evaporator and the dried extract was subjected to initial fractionation on C18 cartridge (Thermo Hypersep C18 10 G) using flash chromatography, with 0, 20, 40, 60, 80 and 100 percent methanol. The first fraction (eluted with 0% MeOH) was found to induce the production of thiolutin and aureothricin, therefore, it was resuspended in 900 µL deionized water and fractionated by HPLC. The HPLC method was same as the one used for fractionation of strain MBN2-2. Fractions 8 and 11 were found to induce the production of both thiolutin and aureothricin and were analysed by LC–MS and NMR.

Hibiscus acid dimethyl ester (**3**): ^1^H NMR [DMSO-*d*_6_]: 6.80 (1H, s), 5.36 (1H, s), 3.76 (3H, s), 3.68 (3H, s), 3.21 (1H, d, *J* = 17.2 Hz), 2.68 (1H, d, *J* = 17.2 Hz). HRESIMS [M + H]^+^ *m/z* 219.0499 (calcd for C_8_H_11_O_7_, 219.099).

Hydroxycitric acid 1,3-dimethyl ester (**4**): ^1^H NMR [DMSO-*d*_6_]: 4.13 (1H, s), 3.62 (3H, s), 3.55 (3H, s), 2.89 (1H, d, *J* = 15.5 Hz), 2.80 (1H, d, *J* = 15.5 Hz). HRESIMS [M + H]^+^ *m/z* 237.0604 (calcd for C_8_H_13_O_8_, 237.0605).

### Inducing production of thiolutin and aureothricin with hydroxycitric acid, citric acid, ammonium *iron* (III) citrate, *iron* (II) sulphate and *iron* (III) chloride

Strain MBN2-2 was grown in 1.5 ml YEME liquid for 24 h. Then from stock solutions of commercially available hydroxycitric acid, ammonium iron (III) citrate, citric acid, iron (II) sulphate, and iron (III) chloride were added to the MBN2-2 culture to give final concentrations of 1000, 750, 500, 250, 100, 50 and 25 µg/ml. Cultures were incubated for seven days at 30 °C with shaking. Each day a 60 µL of extract was taken for antimicrobial assay tests and LC–MS analysis.

### Genomic DNA sequencing and assembly

Genomic DNA from the *Streptomyces* strain MBN2-2 was sequenced by the MicrobesNG DNA Sequencing Facility at the University of Birmingham using a combination of Illumina and Oxford Nanopore Long Reads. Illumina raw reads were trimmed using trim galore [32] and assembled with SPAdes [33] using Unicycler [34] as a SPAdes optimiser. Nanopore raw reads were assembled with Flye [35] and a hybrid assembly was performed with Unicycler [34] and Hybracter [36]. The genome contigs were polished by mapping the Illumina reads back to the assembled contigs with Mira 5.0 [37] and Pilon [38], and joined by manual inspection. The thiolutin/aureothricin biosynthetic gene cluster was identified by analysis of the genome sequence using antiSMASH [39].

### Chrome Azurol S (CAS) assay

The CAS solution was made by the protocol outlined by Alexander and Zuberer [40]. Three separate solutions were prepared. Solution A consisted of 21.9 mg of hexadecyltrimethylammonium bromide (HDTMA) dissolved in 25 ml MiliQ water; Solution B contained 1.5 ml of 1 mM FeCl_3_.6H_2_O dissolved in 10 mM HCl, then mixed with 7.5 ml of 2 mM CAS; Solution C contained 9.76 g (2-(N-morpholino)ethanesulfonic acid) (MES) dissolved in 50 ml MiliQ water with pH adjusted to 5.6 with 1N KOH. Solution B was gradually added to solution A with continuous stirring, followed by addition of solution C. The resulting mixture was adjusted to a final volume of 100 ml with MilliQ water. Just before use, 87.3 mg of sulfosalicylic acid was added to the mixture. The stock solution of 5 mg/ml of the ethylenediaminetetraacetic acid (EDTA), citric acid, hydroxycitric acid, hibiscus acid dimethyl ester, and hydroxycitric acid 1,3-dimethyl ester were prepared in MilliQ water and diluted with CAS solution to achieve final concentrations of 25, 20, 15, 10, 5.0, 2.5, 1.0, 0.5, 0.25, 0.1, 0.05 and 0.01 mg/ml in a 100 µL total reaction volume. MilliQ water and EDTA were used as negative and positive controls, respectively.

### Supplementary Information

Additional file 1.

## Data Availability

All data are included in the main text and its supplementary information file.
