# Supplementary material for: Hibiscus acid and hydroxycitric acid dimethyl esters from Hibiscus flowers induce production of dithiolopyrrolone antibiotics by Streptomyces Strain MBN2-2
Source: Nat Prod Bioprospect. 2024 Jul 3;14(1):40. doi: 10.1007/s13659-024-00460-0 (PMC11219617; doi:10.1007/s13659-024-00460-0)
Supplement: Supplementary file 1 — Additional file 1. [file 13659_2024_460_MOESM1_ESM.docx]

**Supplementary File**

**Hibiscus acid and hydroxycitric acid dimethyl esters from Hibiscus flower induce production of dithiolopyrrolone antibiotics by *Streptomyces* Strain MBN2-2**

Felaine Anne Sumang^1^, Alan Ward^2^, Jeff Errington^1,3^, Yousef Dashti^1,3,*^

^1^ Faculty of Medicine and Health, University of Sydney, Sydney NSW 2015, Australia

^2^ School of Biology, Newcastle University, Newcastle upon Tyne, UK

^3^ Sydney Infectious Diseases Institute, University of Sydney, Sydney NSW 2015, Australia

^*^Corresponding author: [yousef.dashti@sydney.edu.au](mailto:yousef.dashti@sydney.edu.au)

**A**

**B**

**C**

**D**


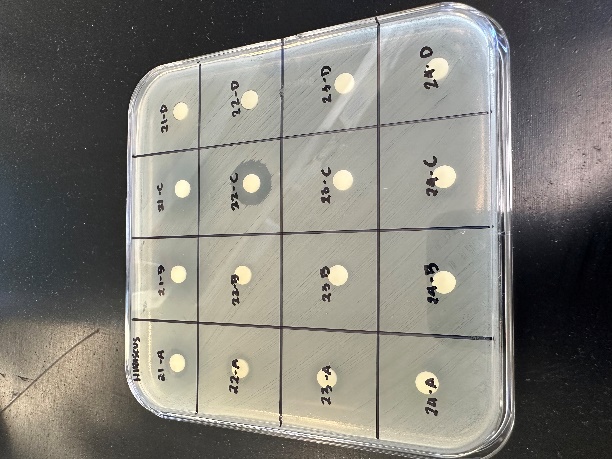


**Figure S1.** Disc diffusion assay of culture supernatant of *Streptomyces* strain MBN2-2 against a lawn of *B.* *subtilis*. Paper discs were treated with culture supernatant of *Streptomyces* strain MBN2-2, supplemented as follows: **A**) nothing added; **B**) DMSO; **C-D**) hibiscus extract at a final concentration of (**C**) 4.5 mg/ml or (**D)** 18 mg/ml.


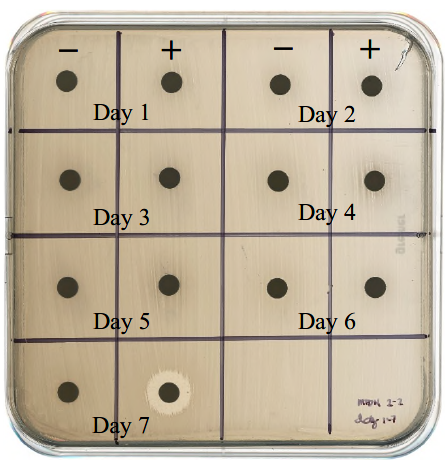


**Figure S2.** Effect of time of culture incubation and presence of hibiscus extract in the culture medium on production of antibiotic activity against *B. subtilis*. *Streptomyces* strain MBN2-2 was cultured in YEME with (+) and without (-) added 4.5 mg/ml of hibiscus extract for 7 days.


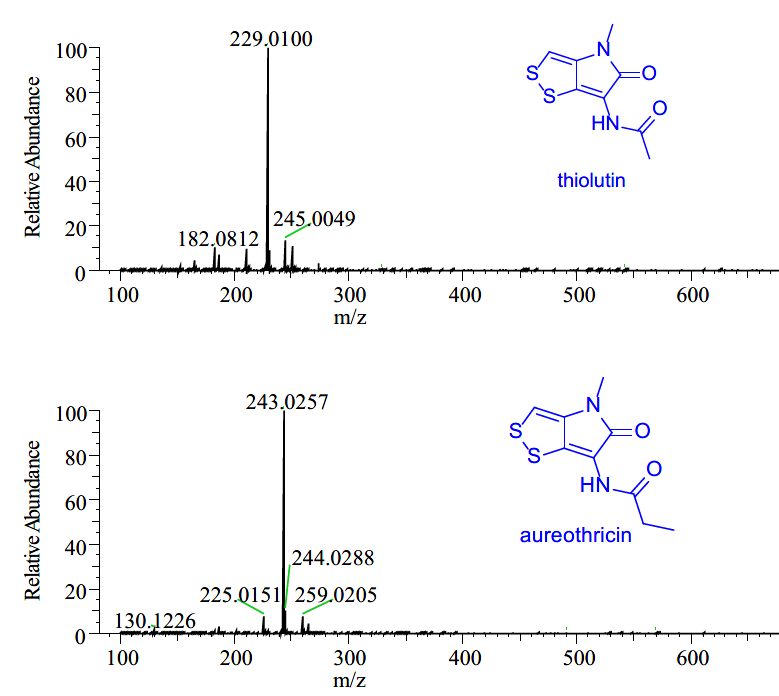


**Figure S3**. High resolution mass spectrometry of induced metabolites thiolutin and aureothricin.


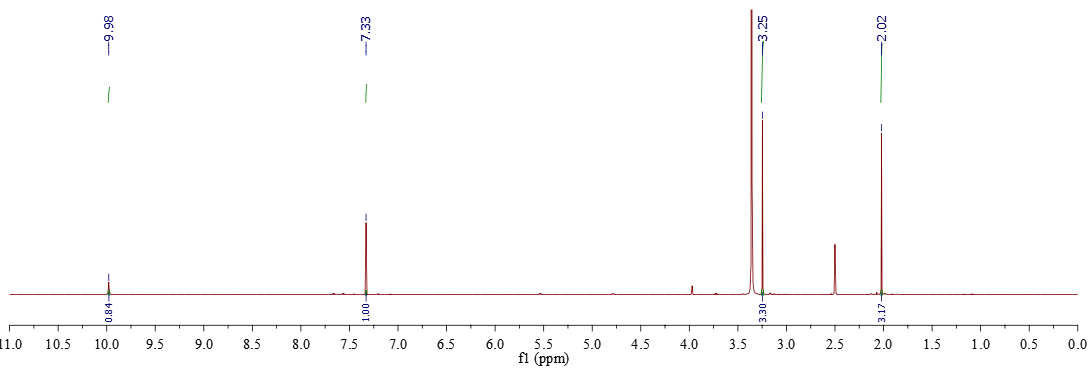


**Figure S4**. ^1^H NMR spectrum of thiolutin in DMSO-*d_6_*.


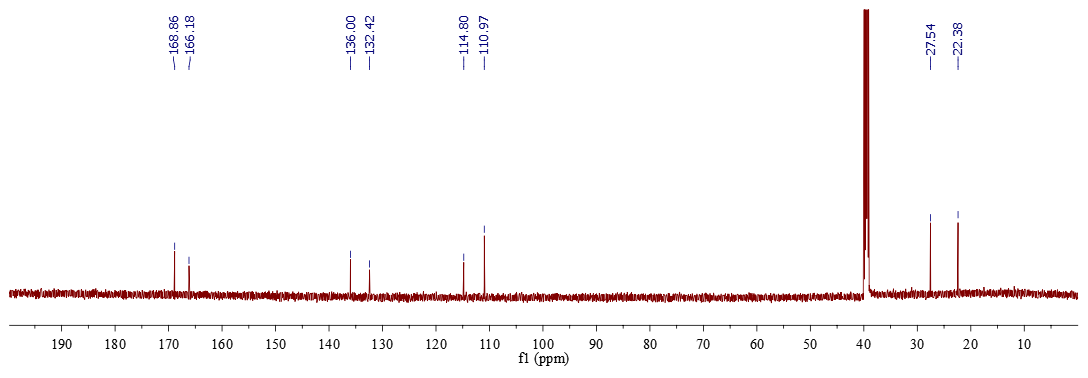


**Figure S5**. ^13^C NMR spectrum of thiolutin in DMSO-*d_6_*.


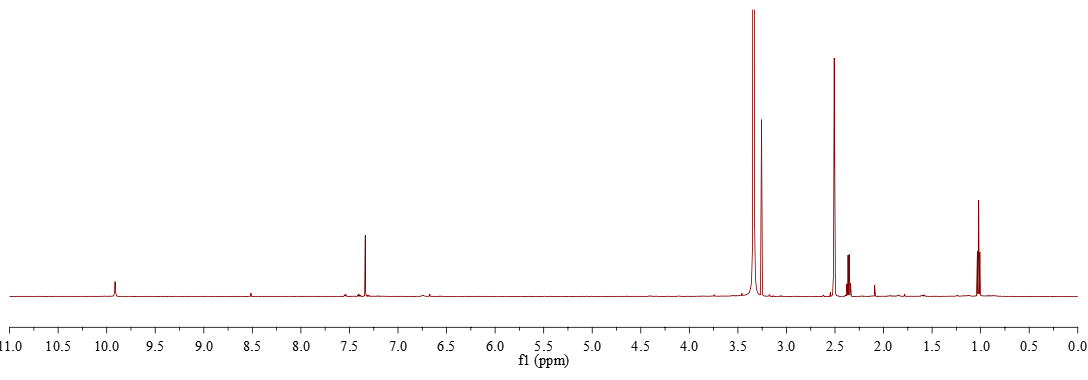


**Figure S6**. ^1^H NMR spectrum of aureothricin in DMSO-*d_6_*.


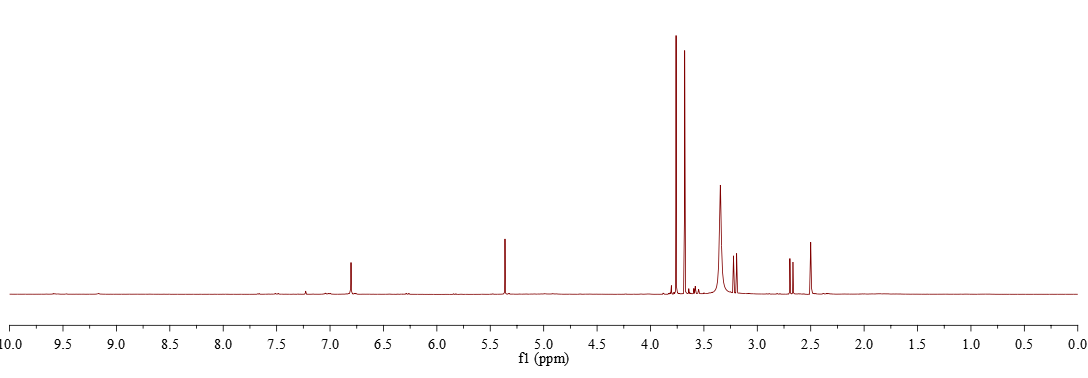


**Figure S7**. ^1^H NMR spectrum of hibiscus acid dimethyl ester in DMSO-*d_6_*.


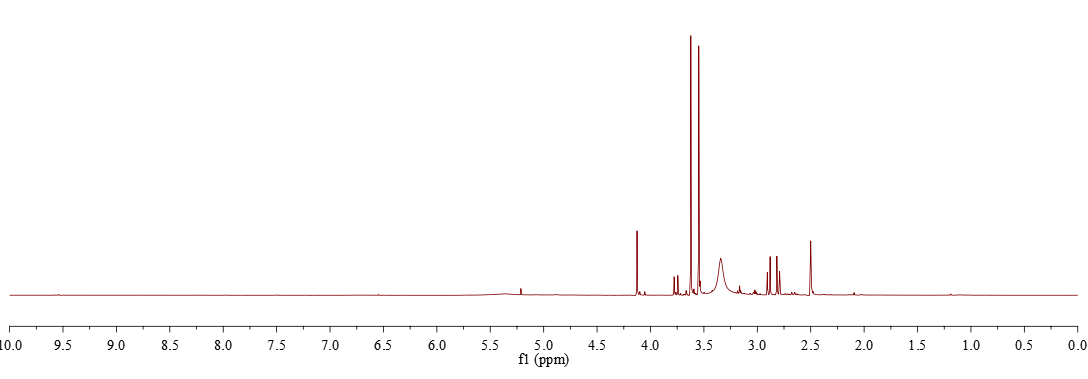


**Figure S8**. ^1^H NMR spectrum of hydroxycitric acid 1,3-dimethyl ester in DMSO-*d_6_*.


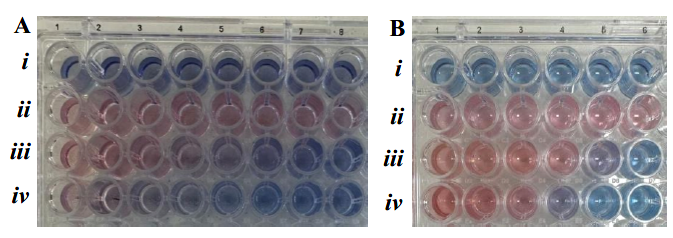


**Figure S9. A)** CAS assay results of ***i*:** milliQ water (negative control), ***ii*:** EDTA (positive control), ***iii*:** citric acid and ***iv*:** hydroxycitric acid, in concentrations 5.0, 2.5, 1.0, 0.5, 0.25, 0.1, 0.05 and 0.01 mg/ml (left to right). **B**) CAS assay results of ***i*:** milliQ water (negative control), ***ii*:** EDTA (positive control), ***iii*:** fraction 8 (dimethyl hibiscus acid), and ***iv*:** fraction 11 (dimethyl hydroxycitric acid), in concentrations 25.0, 20, 15, 10, 5.0, 2.5 mg/ml (left to right).


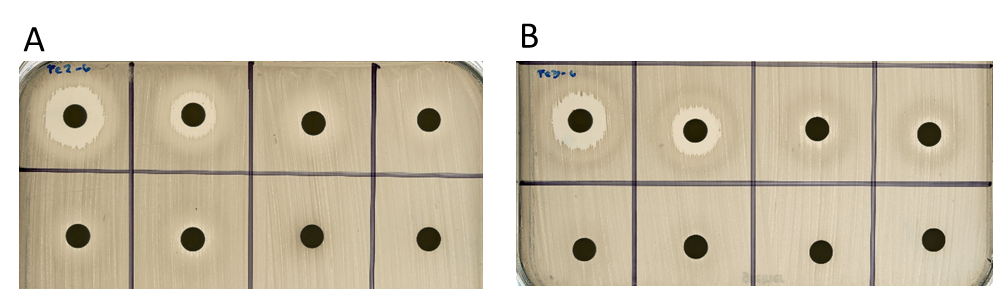


**Figure S10**. Effect of iron concentration on antibiotic production by *Streptomyces* strain MBN2-2. Disc diffusion assay on a lawn of *B. subtilis*. Discs were impregnated with 10 µL of supernatant from a 7 day old culture of *Streptomyces* strain MBN2-2 supplemented with **A)** Fe^2+^, **B)** Fe^3+^, at concentrations of 1, 0.75, 0.5, 0.25, 0.1, 0.05, 0.025 mg/ml (top left to bottom right), with the last a blank control.
